# Pleiotropy among Common Genetic Loci Identified for Cardiometabolic Disorders and C-Reactive Protein

**DOI:** 10.1371/journal.pone.0118859

**Published:** 2015-03-13

**Authors:** Symen Ligthart, Paul S. de Vries, André G. Uitterlinden, Albert Hofman, CHARGE Inflammation working group, Oscar H. Franco, Daniel I. Chasman, Abbas Dehghan

**Affiliations:** 1 Department of Epidemiology, Erasmus University Medical Center, Rotterdam, the Netherlands; 2 Department of Internal Medicine, Erasmus University Medical Center, Rotterdam, the Netherlands; 3 Division of Preventive Medicine, Brigham and Women’s Hospital, Boston, MA, United States of America; University of North Carolina, UNITED STATES

## Abstract

Pleiotropic genetic variants have independent effects on different phenotypes. C-reactive protein (CRP) is associated with several cardiometabolic phenotypes. Shared genetic backgrounds may partially underlie these associations. We conducted a genome-wide analysis to identify the shared genetic background of inflammation and cardiometabolic phenotypes using published genome-wide association studies (GWAS). We also evaluated whether the pleiotropic effects of such loci were biological or mediated in nature. First, we examined whether 283 common variants identified for 10 cardiometabolic phenotypes in GWAS are associated with CRP level. Second, we tested whether 18 variants identified for serum CRP are associated with 10 cardiometabolic phenotypes. We used a Bonferroni corrected p-value of 1.1×10^-04^ (0.05/463) as a threshold of significance. We evaluated the independent pleiotropic effect on both phenotypes using individual level data from the Women Genome Health Study. Evaluating the genetic overlap between inflammation and cardiometabolic phenotypes, we found 13 pleiotropic regions. Additional analyses showed that 6 regions (*APOC1*, *HNF1A*, *IL6R*, *PPP1R3B*, *HNF4A* and *IL1F10*) appeared to have a pleiotropic effect on CRP independent of the effects on the cardiometabolic phenotypes. These included loci where individuals carrying the risk allele for CRP encounter higher lipid levels and risk of type 2 diabetes. In addition, 5 regions (*GCKR*, *PABPC4*, *BCL7B*, *FTO* and *TMEM18*) had an effect on CRP largely mediated through the cardiometabolic phenotypes. In conclusion, our results show genetic pleiotropy among inflammation and cardiometabolic phenotypes. In addition to reverse causation, our data suggests that pleiotropic genetic variants partially underlie the association between CRP and cardiometabolic phenotypes.

## Introduction

The risk of cardiometabolic diseases, the world’s leading cause of mortality, is higher in people with elevated levels of systemic inflammation, independent of traditional cardiometabolic risk factors [1]. Elevated levels of C-reactive protein (CRP), as a measurement of systemic inflammation, are associated with hypertension [2], type 2 diabetes (T2D) [3,4], coronary artery disease (CAD) [1,5,6], stroke [7,8], peripheral artery disease [9], and mortality [10]. Although observational data suggest a link between CRP and cardiometabolic phenotypes, Mendelian randomization approaches have provided evidence against a causal link between CRP and these cardiometabolic phenotypes [11–14].

Genome-wide association studies (GWAS) have discovered multiple single-nucleotide polymorphisms (SNPs) associated with inflammatory markers including CRP and different cardiometabolic phenotypes including T2D, coronary artery disease (CAD), lipids and hypertension [15–21]. From these GWAS we already learned that several genes, such as *IL6R*, *APOC1*, *GCKR* and *HNF1A*, are associated both with systemic inflammation and cardiometabolic phenotypes such as CAD, lipids and diabetes [15,17,21,22]. This phenomenon of one genetic locus affecting more than one phenotype is called genetic “pleiotropy”. In general, two types of pleiotropy can be defined. As previously defined by Solovieff et al., “biological pleiotropy” refers to a gene that has independent biological effects on more than one phenotype, and “mediated pleiotropy” refers to the situation where the genetic effect on phenotype B is mediated by phenotype A that is causally related to phenotype B [23]. Although both types of pleiotropy are interesting, only biological pleiotropy refers to the genuine pleiotropy where the effect of the genetic variant on two or more phenotypes is independent.

We hypothesize that in addition to reverse causation, genetic loci with pleiotropic effects may underlie the association between CRP and cardiometabolic phenotypes. To this end, we applied a simple and robust approach to point out these pleiotropic genetic variants [24]. First, we examined whether common variants identified for cardiometabolic phenotypes are associated with serum CRP levels as a measure of systemic inflammation. Second, we conversely examined whether variants so far identified for serum CRP associate with cardiometabolic phenotypes. In addition, we adjusted the association between the SNP and CRP for the cardiometabolic phenotypes and vice versa to distinguish a genuine biological pleiotropic effect from mediated pleiotropy.

## Materials and Methods

### Study design

To examine the overlap between genes for inflammation and cardio-metabolic disorders we collected GWAS meta-analyses data from published GWAS on cardiometabolic phenotypes and CRP [15–17,19,22]. These GWAS are mainly conducted in individuals from European ancestry (Table 1). We tested the genetic association of cardiometabolic SNPs with systemic inflammation using the largest published GWAS meta-analysis on CRP levels from the CHARGE (the Cohorts for Heart and Aging Research in Genomic Epidemiology) inflammation working group [22]. Testing the genetic association of the CRP SNPs with 10 cardiometabolic phenotypes we used the recent GWAS data from the following consortia: Coronary Artery Disease Genome-wide Replication and Meta-analysis plus the Coronary Artery Disease, CARDIoGRAMplusC4D [15], International Consortium for Blood Pressure, ICBP [16], the Meta-Analyses of Glucose and Insulin-related traits Consortium, MAGIC [17,18], DIAbetes Genetics Replication And Meta-analysis, DIAGRAM [19], The Genetic Investigation of Anthropometric Traits, GIANT [20] and Global Lipids Genetic Consortium, GLGC [21]. Additionally, we carried out analyses in a population based cohort study to explore the type of pleiotropy of the overlapping SNPs.

**Table 1:** Genome-wide association studies of cardiometabolic phenotypes and inflammation.

Consortium	Phenotype	Sample size	No. of Studies
GIANT [20]	BMI	249,796	62
GLGC [21]	HDLC, LDLC, TG, TC	99,900	46
ICBP [16]	SBP, DBP	69,395	29
MAGIC [19]	FG, FI	133,010	32
DIAGRAM [18]	T2D	149,821	38
CARDIoGRAMplusC4D [15]	CAD	194,427	49
CHARGE inflammation [22]	CRP	82,725	25

Abbreviations: BMI, body mass index; CAD, coronary artery disease; CARDIoGRAMplusC4D, Coronary Artery Disease Genome-wide Replication and Meta-Analysis plus Coronary Artery Disease Genetics Consortium; CHARGE, Cohorts for Heart and Aging Research in Genomic Epidemiology; CRP, c-reactive protein; DBP, diastolic blood pressure; DIAGRAM, DIAbetes Genetics Replication And Meta-analysis; FG, fasting glucose; FI, fasting insulin; GIANT, Genetic Investigation of ANthropometric Traits; GLGC, Global Lipids Genetic Consortium; HDLC, HDL-cholesterol; ICBP, International Consortium for Blood Pressure; LDLC, LDL-cholesterol; MAGIC, Meta-Analyses of Glucose and Insulin-related traits Consortium; SBP, systolic blood pressure; T2D, type 2 diabetes; TC, total cholesterol; TG, triglycerides.

### Cardiometabolic SNPs and association with CRP

We first compiled a list of genome-wide significant SNPs (p-value < 5×10^-8^) previously identified in large GWAS on cardiometabolic traits to test the genetic association in the CRP GWAS. The following cardiometabolic traits were included to generate the SNP list: coronary artery disease (51 SNPs in CARDIOGRAMplusC4D, n = 130,681 with 63,746 cases) [15]; blood pressure (29 SNPs in ICBP, n = 69,395) [16]; fasting glucose, fasting insulin (53 SNPs in MAGIC, n = 133,010) [17,18]; type 2 diabetes (55 SNPs in DIAGRAM, n = 149,821 with 34,840 cases) [19]; body-mass index (38 SNPs in GIANT, n = 123,865) [20]; LDL cholesterol, HDL cholesterol, triglycerides and total cholesterol (102 loci in GLGC, n = 100,184) [21]. When the SNP was not available in the CRP GWAS, we searched for a proxy with an *r* ^*2*^ > 0.8. For 6 SNPs, this was not possible. LD-based pruning was performed (r^2^ threshold of 0.3) using HapMap LD information to make sure that independent SNPs were included in the analysis [25]. The SNP with the lowest p-value in one of the cardiometabolic GWAS was chosen. The final list included 283 independent SNPs that are genome-wide significantly associated with one or more cardiometabolic phenotypes.

### CRP SNPs and association with cardiometabolic phenotypes

We used the publically available GWAS meta-analyses data to test whether any of the 18 independent genome-wide significant SNPs identified in the CRP GWAS were associated with the following cardiometabolic phenotypes: LDL cholesterol, HDL cholesterol, triglycerides and total cholesterol (GLGC); body mass index (GIANT); systolic blood pressure (ICBP); coronary artery disease (CARDIoGRAMplusC4D consortium); fasting glucose and fasting insulin (MAGIC); type 2 diabetes (DIAGRAM). All available GWASs provided p-values for all 18 CRP SNPs, except the GWAS on CAD and the glycemic phenotypes which were based on a custom chip array (Metabochip array [26]) containing 79,000 SNPs and this array did not include 8 of the CRP SNPs. For the SNPs that were not on the Metabochip, we used for fasting glucose and fasting insulin the previous GWAS published by Dupuis et al. [17], for type 2 diabetes only the stage 1 GWAS including all HapMap SNPs [19] and for CAD we used the summary data from the CARDIoGRAM meta-analysis only [27].

### CRP and cardiometabolic measures

Coronary artery diseases was defined in the CARDIoGRAMplusC4D consortium using standard criteria for myocardial infarction or coronary artery disease namely symptoms of angina pectoris, previous myocardial infarction or cardiac intervention [15]. Hypertension was defined in the ICBP consortium as systolic blood pressure ≥ 140 mmHg or diastolic blood pressure ≤ 90 mmHg [16]. Fasting glucose and fasting insulin were measured in MAGIC using standard laboratory techniques [17]. Type 2 diabetes was in the DIAGRAM consortium defined as fasting plasma glucose level ≥ 7.0 mmol/l or non-fasting glucose plasma level ≥ 11.0 mmol/l and/or treatment with oral antidiabetic medication or insulin [19]. LDL cholesterol, HDL cholesterol, triglycerides and total cholesterol were measured in the GLGC using standard laboratory techniques [21].

We used the discovery panel of the recently published GWAS meta-analysis on serum CRP (CHARGE Inflammation) [22]. The meta-analysis included 15 studies in the discovery panel (n = 65,000). CRP was natural log-transformed (lnCRP) and effects represented a 1-unit change in lnCRP per copy increase in risk allele.

### Statistical methods

In this study we evaluated 463 possible SNP-phenotype associations including 283 independent cardiometabolic SNPs in the CRP GWAS and 18 independent CRP SNPs in 10 different cardiometabolic GWAS. To address the issue of multiple testing we used a Bonferroni corrected alpha of 1.1×10^-4^ (0.05/463 tests) as a robust threshold for a significant association between the SNP and the phenotype in our study [28].

In a quantile-quantile (Q-Q) plot, a nominal probability distribution is compared against an empirical distribution. In the scenario that the nominal p-values form a straight line on a Q-Q plot when they are plotted against the empirical distribution, all relations are null. When the observed distribution is deflected to the left from the uniform null distribution, lower p-values are observed compared to that expected by chance (enrichment). We used QQ-plots to evaluate whether SNPs that are genome-wide significant associated with the cardiometabolic phenotype, were in the CRP GWAS distributed differently from what is expected under the null-hypothesis. Vice versa, we evaluated whether genes identified for CRP were in the cardiometabolic GWAS distributed differently from what is expected under the null-hypothesis. We used Fisher’s combined probability test to test for significant enrichment in the QQ-plots [29].

### Evaluation of the type of pleiotropy

To clarify the type of genetic pleiotropy (biological or mediated), we performed additional analyses in the Women’s Genome Health Study (WGHS) including 23,294 women [30]. In the first model, we analyzed the age-adjusted association between CRP (dependent variable) and the lead SNP for CRP in the pleiotropic regions. To examine whether the association is independent of cardiometabolic traits we further adjusted this association for BMI, lipid levels (HDL-cholesterol, LDL-cholesterol, triglycerides and total cholesterol) and HbA1C. We used HbA1C as a proxy for glycemic metabolism given the fact that glycated hemoglobin is an acceptable marker of average blood glucose level in the last 2–3 months [31]. In addition, we adjusted the association for age and in a stepwise manner we added lipids, BMI and HbA1C to the model to evaluate the different effects of the phenotypes on the association. Last, we analyzed the association between the pleiotropic SNP and the associated cardiometabolic phenotypes unadjusted and adjusted for CRP. As we tested 43 SNP-phenotype associations in the WGHS, we used a Bonferroni corrected alpha of 1.2×10^-03^ as a threshold of study-wide significance. All regression analyses were carried out in the statistical software R version 2.15.3 [32].

### Women’s Genome Health Study (WGHS)

The WGHS is a prospective cohort of female North American health care professionals representing participants in the Women’s Health Study who provided a blood sample at baseline and consent for blood-based analyses. Participants were 45 or older at enrollment and free of cardiovascular disease, cancer or other major chronic illness. The current data are derived from 23,294 WGHS participants with whole genome genetic data and verified self-reported, European ancestry. The study protocol was approved by the institutional review board of the Brigham and Women’s Hospital (Boston, MA, USA). All participants provided written informed consent to participate in the study.

### Covariates WGHS

BMI (weight in kilograms divided by height in meters squared) was calculated from responses to the baseline questionnaire. All baseline blood samples underwent measurement for high-sensitivity C-reactive protein (hsCRP) via a validated immunoturbidometric method (Denka Seiken, Tokyo, Japan). Concentrations of total cholesterol (TC) and HDL-C were measured enzymatically on a Hitachi 911 autoanalyzer (Roche Diagnostics) with day-to-day reproducibility of 1.36% and 1.07% for TC concentrations of 129.8 and 277.2 mg/dL, respectively, (throughout this report, concentrations and units given are those reported in the referenced sources) and of 1.98% and 2.68% for HDL-C concentrations of 35 and 55 mg/dL, respectively. LDL-C was determined directly (Genzyme) with reproducibility of 2.16% and 1.98% for concentrations of 76.2 and 148.7 mg/dL, respectively. Triglycerides were measured enzymatically, with correction for endogenous glycerol, using a Hitachi 917 analyzer and reagents and calibrators from Roche Diagnostics; reproducibility was 1.52% and 1.49% for triglyceride concentrations of 82.5 and 178.8 mg/dL, respectively. Hemoglobin A1c was measured using turbidimetric immunoinhibition directly from packed red blood cells (Roche Diagnostics) with reproducibility of 3.63% and 3.77% at levels of 5.2% and 8.8%, respectively. A total of 22,773 participants with genotyped and covariates available were included in this study.

### Genotyping WGHS

DNA extracted from the baseline blood samples underwent SNP genotyping via the Illumina Infinium II assay for querying of a genome-wide set of SNPs from the Illumina HumanHap300 Duo “+” platform. This panel including the standard content of approximately 318,237 SNPs covering the entire genome from the HumanHap300 panel with an additional focused panel of 45,571 SNPs selected to enhance coverage of cardiovascular candidate genes and SNPs with suspected functional consequences. For the current analysis, all samples had successful genotype information for >98% of the SNPs, while all SNPs had successful genotype information for >90% of the samples. SNPs with significance p <10^-6^ for deviations from Hardy-Weinberg equilibrium were excluded from analysis. Self-reported European ancestry was confirmed in the 23,294 samples on the basis of a principal component analysis using PLINK among 1,443 ancestry informative SNPs selected for Fst >0.4 in the HapMap2. In total, 339,875 genotyped SNPs passing the criteria for inclusion also had minor allele frequency at least 1 percent. On the basis of linkage disequilibrium relationships in the HapMap (release 22), genotypes for a total of 2,621,896 SNPs were imputed from the 23,294 samples passing the quality criteria using Mach v. 1.0.16.

### Pathway analysis

Pathway analysis was performed on the pleiotropic loci that we identified using Ingenuity Pathway Analysis software tool (IPA Ingenuity Systems). The Ingenuity Knowledge Base (including only genes) was used as a reference set and we considered molecules and/or direct and indirect relationships. The confidence filter was set to experimentally observed or high (predicted). Pathways were generated with a maximum size of 35 genes and we allowed up to 25 pathways. The significance p-value associated with enrichment of pathways was calculated using the right-tailed Fisher’s exact test, considering the number of query molecules that participate in that pathway and the total number of molecules that are known to be associated with that pathway in the reference set. A False Discovery Rate of five percent was used as a threshold of significance using the Benjamini-Hochberg method.

## Results

### Cardiometabolic SNPs in CRP GWAS

**Fig 1:**
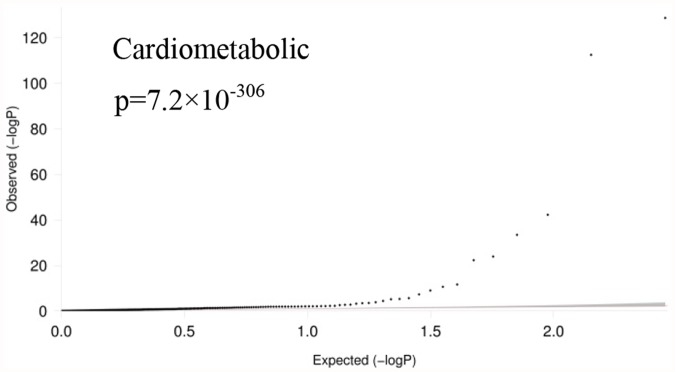
Quantile-quantile plot of cardiometabolic SNPs in CRP GWAS. QQ-plot was used to evaluate whether SNPs that are genome-wide significant associated with the cardiometabolic phenotypes, were in the CRP GWAS distributed differently from what is expected under the null-hypothesis. The observed p-values (dotted line) for the phenotypes deviated significantly leftwards indicating that these p-values are smaller than expected under null hypothesis.

First, we used QQ-plots to evaluate whether the p-values for the associations of the 283 cardiometabolic SNPs with serum CRP are distributed differently from what is expected under the null hypothesis in each trait group. As depicted in Fig. 1, the leftward deviation of the dotted lines in the QQ-plots shows that the 283 SNPs known for cardiometabolic phenotypes to have p-values smaller than expected under the null hypothesis in the CRP GWAS (p-value: 7.2×10^-306^).

A total of 19 SNPs out of 283 independent cardiometabolic SNPs (6.7%) were associated with CRP after correction for multiple testing (p-value threshold 1.1×10^-04^). These 19 SNPs were located within or close to 12 different genes *APOC1*, *HNF1A*, *GCKR*, *IL6R*, *PPP1R3B*, *HNF4A*, *PABPC4*, *BCL7B*, *FTO*, *TMEM18*, *PLTP* and *MC4R*. Table 2 shows the SNPs with the lowest p-values in the 12 pleiotropic loci based on the CRP GWAS, i.e. the lowest p-value in that genomic locus. The eight SNPs in Table 2 with the lowest p-value were already known to be associated with CRP based on the recent CRP GWAS [22]. The next four SNPs were not identified in the genome-wide association study of CRP. The first novel association was rs1558902 with a p-value of 2.2×10^-6^. This SNP is located in the first intron of the *FTO* gene on chromosome 16. The second novel signal was the SNP rs2867125 which is located on chromosome 2, near 46kb downstream of *TMEM18*. This SNP had a p-value of 5.0×10^-6^ in the CRP meta-analysis. The third association was with rs6065906 which is located on chromosome 20, near the *PLTP* and *PCIF1* gene (p-value = 6.7×10^-6^). The last finding was rs571312 which is located 2 Mb upstream of the *MC4R* gene on chromosome 18 (p-value = 3.8×10^-5^).

**Table 2:** The association of known loci for cardiometabolic traits with serum CRP.

SNP	Band	A1/A2^a^	Effect^b^ (SE)	P-value	Gene	Phenotypes (effect direction)	Top-SNP^c^ (*r* ^*2*^; P-value)
rs4420638	19q13.32	A/G	0.240 (0.010)	2.1×10^-129^	*APOC1*	TG(-), TC(-), HDLC(+), LDLC(-)	The same
rs1169288	12q24.31	A/C	0.152 (0.007)	3.3×10^-113^	*HNF1A*	TC(-), LDLC(-), T2D(+)	rs1183910(0.96; 3.3×10^-113^)
rs1260326	2p23.3	T/C	0.089 (0.007)	5.5×10^-43^	*GCKR*	TC(+), TG(+), FG(-), FI(-)	The same
rs4845625	1q21.3	T/C	0.062 (0.006)	4.8×10^-23^	*IL6R*	CAD(+)	rs4129267(0.519; 1.1×10^-47^)
rs9987289	8p23.1	G/A	0.079 (0.011)	2.3×10^-12^	*PPP1R3B*	TC(+), HDLC(+), LDLC(+), FI(-), FG(-)	The same
rs1800961	20q13.12	C/T	0.120 (0.018)	2.3×10^-11^	*HNF4A*	TC(+), HDLC(+), T2D(+)	The same
rs4660293	1p32.4	G/A	0.044 (0.007)	9.9×10^-10^	*PABPC4*	HDLC(-)	rs12037222(0.955; 4.5×10^-10^)
rs17145738	7q11.23	C/T	0.054 (0.010)	4.7×10^-8^	*BCL7B*	HDLC(-), TG(+)	rs13233571(1.00; 2.8×10^-8^)
rs1558902	16q12.2	A/T	0.032 (0.007)	2.2×10^-6^	*FTO*	BMI(+), T2D(+)	The same
rs2867125	2p25.3	C/T	0.038 (0.008)	5.0×10^-6^	*TMEM18*	BMI(+)	rs10189761(0.929; 1.2×10^-6^)
rs6065906	20q13.12	T/C	0.036 (0.008)	6.7×10^-6^	*PLTP*	HDLC(+), TG(-)	rs6073972(1.00; 2.9×10^-6^)
rs571312	18q22	A/C	0.033 (0.008)	3.8×10^-5^	*MC4R*	BMI(+)	The same

^a^ Effect represents 1-unit change in the natural log-transformed CRP (mg/L) per copy increase in the risk allele. SE, standard error.

^b^ A1 and A2 represent respectively the risk allele and non-risk allele.

^c^ Top-SNP represents the SNP with the lowest p-value in the genomic region in the CRP meta-analysis. If the top-SNP is “The same”, the top SNP for the cardiometabolic traits is the same as the SNP with the lowest p-value in the CRP meta-analysis.

*Note*: p-value ≤ 1.1×10^-4^ is considered as study-wide significant (0.05/463)

Abbreviations: BMI, body mass index; CAD, coronary artery disease; FG, fasting glucose; FI, fasting insulin; HDLC, high-density lipoprotein cholesterol; LDL, low-density lipoprotein cholesterol; T2D, type 2 diabetes; TC, total cholesterol; TG, triglycerides.

Among the associated SNPs, we observed many SNPs with different directions of effect on the cardiometabolic phenotypes and CRP than one would expect based on the association of CRP and these phenotypes in observational data. As an example, the A-allele of the SNP rs4420638 in the *APOC1* locus increases serum CRP levels. However, this allele is associated with a decrease in the level of total cholesterol, LDL-cholesterol and triglycerides. We also observed such effects for the G-allele of the SNP rs1183910 in the *HNF1A* locus. This allele increases serum CRP levels and is associated with a decline in total cholesterol and LDL-cholesterol.

Out of the 12 pleiotropic loci, 6 loci had the same lead SNP in both the CRP and one or more of the cardiometabolic GWAS. In the other 6 loci the lead SNPs were different between the CRP GWAS and the cardiometabolic GWAS. However, in the majority of these loci the lead SNPs of the cardiometabolic GWAS were in high LD (*r* ^2^ > 0.8) with the lead SNP in the CRP GWAS. In the *IL6R* locus we observed the lowest LD between the top hit in the CRP GWAS and the CAD GWAS (*r* ^2^ = 0.52).

### CRP SNPs in cardiometabolic GWAS

**Fig 2:**
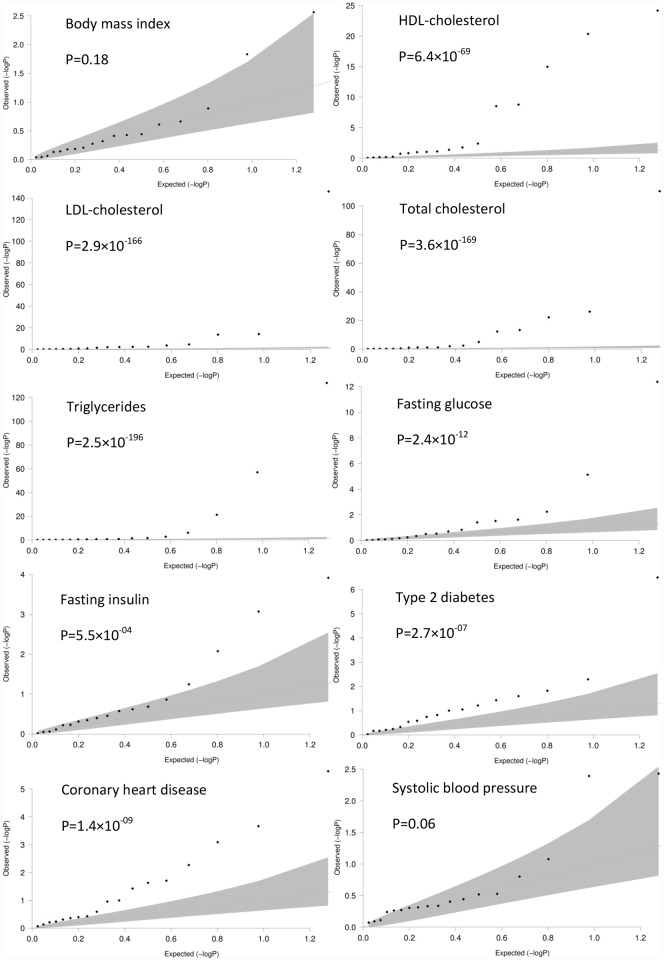
Quantile-quantile plots of CRP SNPs in cardiometabolic GWAS. QQ-plots were used to evaluate whether SNPs that are genome-wide significant associated with CRP, were in the cardiometabolic GWAS distributed differently from what is expected under the null-hypothesis. The observed p-values (dotted line) for the phenotypes HDL-cholesterol, fasting glucose, type 2 diabetes and coronary artery disease deviated significantly leftwards indicating that these p-values are smaller than expected under null hypothesis.

We used the same QQ-plots as described previously to evaluate whether the association p-values for the 18 CRP SNPs are distributed differently from what is expected under the null hypothesis in the different cardiometabolic GWAS. As depicted by the leftward deviation of the dotted lines in the QQ-plots for CAD (p = 1.4×10^-09^), the cholesterol phenotypes (HDL-cholesterol, p = 6.4×10^-69^; LDL-cholesterol, p = 2.9×10^-166^; total cholesterol, p = 3.6×10^-169^ and triglycerides, p = 2.5×10^-196^) and the glycemic phenotypes (fasting glucose, p = 2.4×10^-12^ and fasting insulin 5.5×10^-04^), the p-values for the association between the 18 CRP SNPs and these phenotypes are significantly smaller than expected under the null hypothesis (Fig. 2). We did not observe such a significant deviation in the QQ-plots of BMI (p = 0.18) and SBP (p = 0.06).

**Fig 3:**
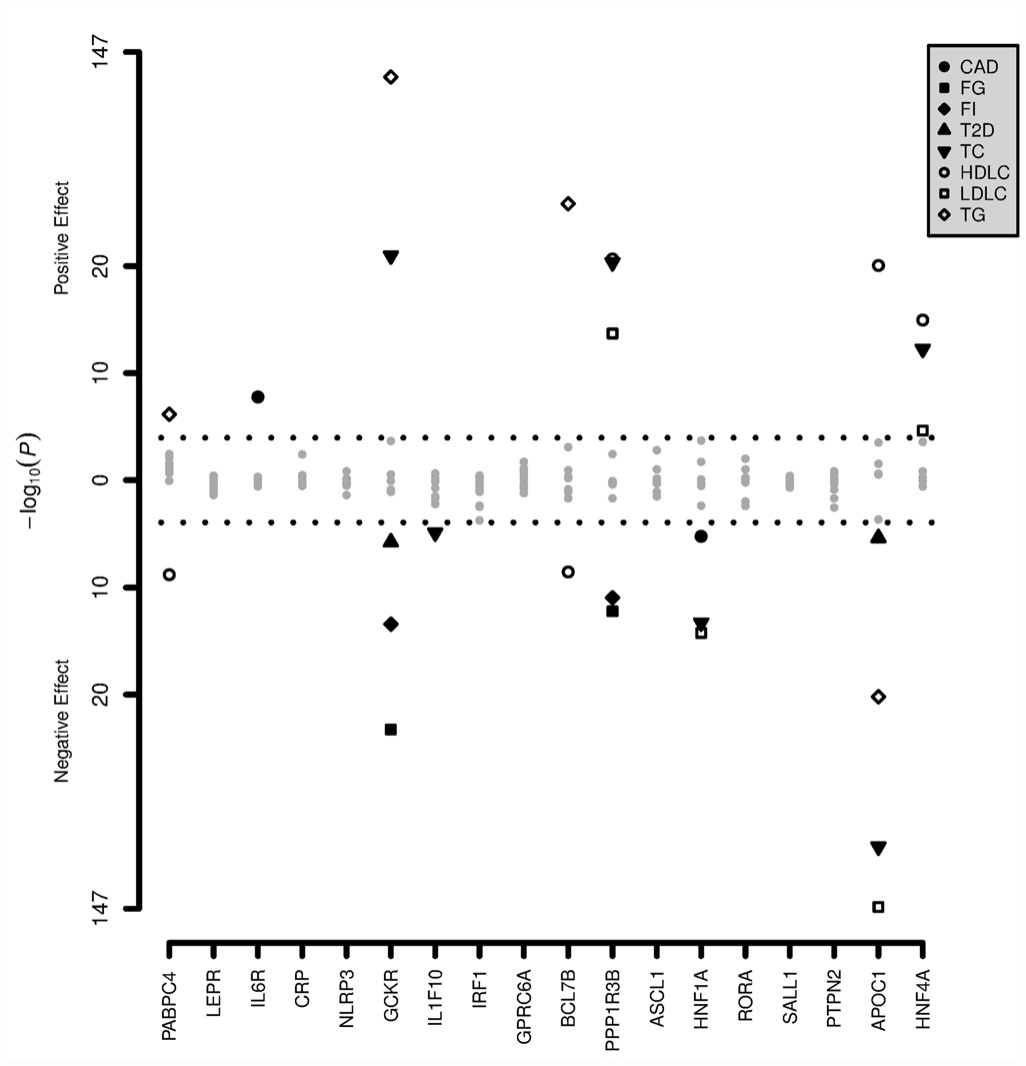
P-values for the associations of the 18 CRP SNPs with different cardiometabolic phenotypes. P-values for the associations between the 18 CRP SNPs and BMI, lipids, glycemic phenotypes, SBP and coronary artery disease. The genes on the x-axis represent the genes in which the CRP SNPs are located or closest by. The numbers on the y-axis indicate the p-values of the association between the SNPs and the cardiometabolic phenotypes. Significant associations are colored as depicted in the figure legend. For BMI and SBP, no significant associations were observed. CAD, coronary artery disease; FG, fasting glucose; FI, fasting insulin; HDLC, HDL-cholesterol; LDLC, LDL-cholesterol; T2D, type 2 diabetes; TC, total cholesterol; TG, Triglycerides.

Results of the association of the 18 genome-wide significant associations with CRP-level are depicted in Fig. 3 (S1 and S2 tables). We observed 9 associations with one or more of the 10 cardiometabolic phenotypes close to or within the genes *APOC1*, *HNF1A*, *IL6R*, *GCKR*, *IL1F10*, *PPP1R3B*, *HNF4A*, *PABPC* and *BCL7B* (p-value < 1.1×10^-4^). Only 1 gene (*IL1F10*) was not identified in the previous analysis where we tested the association between the cardiometabolic SNPs and CRP. Among all 9 associations, we found three associations that are not reported in the GWAS for that specific phenotype. The first was rs1183910 with CAD (p-value 5.6×10^-6^). This SNP is located in the first intron of the *HNF1A* gene on chromosome 12. The second was rs6734238 with total cholesterol (p-value 1.16×10^-5^). This SNP is located on chromosome 2, nearby the *IL1F10* gene and other interleukin 1 family genes. The third was rs4420638 with T2D (p-value 4.0x10^-6^) nearby the *APOC1* gene on chromosome 19.

Comparable with the previous associations results, we observed many different direction of effects. For instance, the A-allele of the SNP rs4420638 in the *APOC1* locus increases serum CRP levels and is associated with a lower risk of type 2 diabetes. Furthermore, the G-allele of the SNP rs1183910 in the *HNF1A* locus increases serum CRP levels and is associated with a lower risk of coronary artery disease.

### Exploring the type of pleiotropy

**Fig 4:**
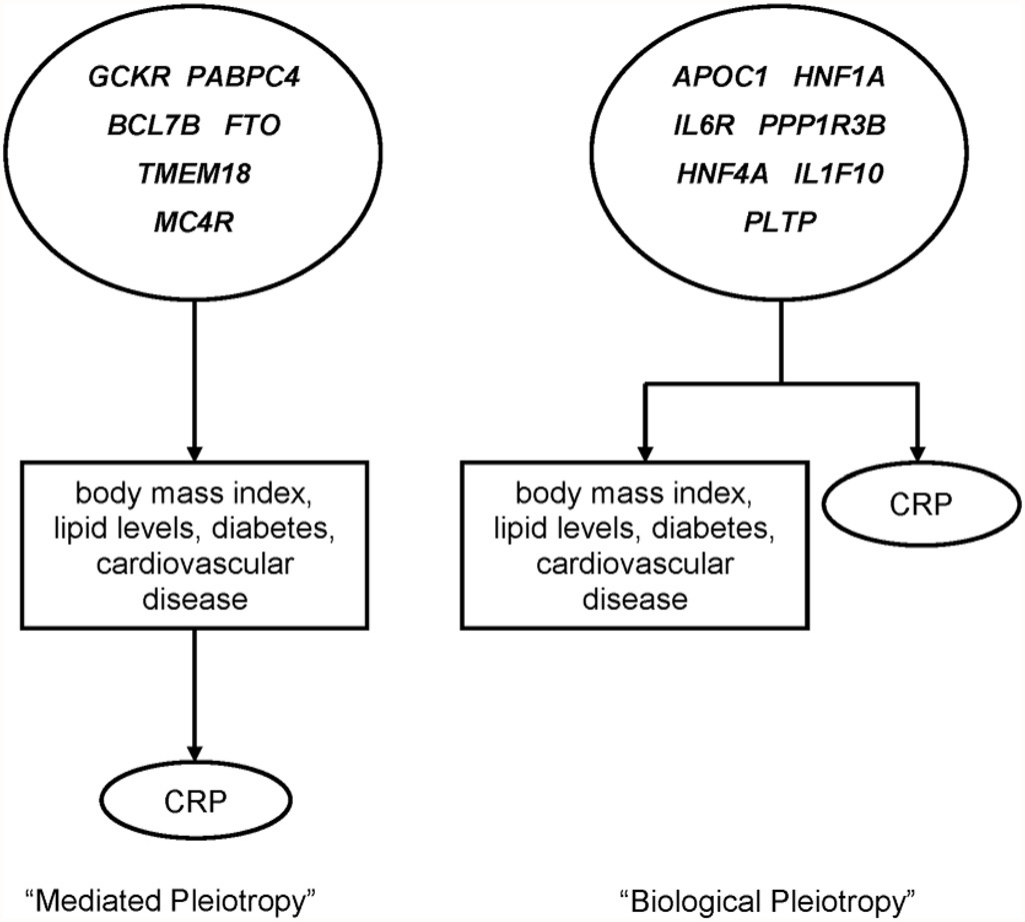
Biological and mediated pleiotropy of overlapping loci among inflammation and cardiometabolic phenotypes. Overlapping loci among inflammation and cardiometabolic phenotypes and type of pleiotropy according to the additional analyses. We identified six overlapping loci with mediated pleiotropic effects on CRP (left) and seven with a biological pleiotropic effect (right).

We observed a total number of 13 genetic regions with pleiotropic effects on CRP and cardiometabolic phenotypes: 12 regions identified in the first step testing the cardiometabolic SNPs with CRP and 1 additional region identified in the second step testing the associations of the CRP SNPs with the cardiometabolic phenotypes. Table 3 shows the unadjusted and adjusted associations between the 13 overlapping SNPs and CRP-level using individual level data from the WGHS. There was no significant association in the WGHS between the SNPs located near *PLTP* and *MC4R* and CRP after correction for multiple testing. The effect sizes of the genetic loci in or near the genes *APOC1*, *HNF1A*, *IL6R*, *PPP1R3B*, *HNF4A* and *IL1F10* did not diminish substantially after adjustment for BMI, cholesterol levels and HbA1C suggesting biological pleiotropy. For *BCL7B*, *FTO* and *TMEM18* the effect sizes decreased considerably implying mediated pleiotropy. The estimate of the association between rs1260326 (*GCKR*) and CRP decreased substantially after adjustment but was still strongly associated. We observed the same scenario for the association between rs4660293 (*PABPC4*) and CRP. When we added the phenotypes in a stepwise manner to the model, we observed for the mediated pleiotropic loci *FTO* and *TMEM18* that the effect was mainly mediated through BMI (S3 Table). For *BCL7B* and *PABPC4*, lipids appeared to be the most important mediators. Fig. 4 shows graphically the biological and mediated pleiotropic effects.

**Table 3:** Pleiotropic SNPs and their association with CRP.

		* MODEL 1* ^*a*^			* MODEL 2* ^b^				
SNP	CHR	beta	se	pval	beta	se	pval	gene	pleiotropy^c^
rs4420638	19	0.269	0.019	4.4×10^-47^	0.272	0.016	1.7×10^-65^	*APOC1*	B
rs1169288	12	0.165	0.012	2.3×10^-43^	0.160	0.010	4.0×10^-56^	*HNF1A*	B
rs1260326	2	0.110	0.011	1.6×10^-22^	0.073	0.010	3.4×10^-14^	*GCKR*	M
rs4845625	1	0.067	0.011	2.0×10^-9^	0.065	0.009	8.8×10^-12^	*IL6R*	B
rs9987289	8	0.076	0.019	4.5×10^-5^	0.086	0.016	1.5×10^-7^	*PPP1R3B*	B
rs1800961	20	0.146	0.033	8.4×10^-6^	0.141	0.028	4.7×10^-7^	*HNF4A*	B
rs4660293	1	0.048	0.013	1.9×10^-4^	0.036	0.011	1.2×10^-3^	*PABPC4*	M
rs17145738	7	0.075	0.017	1.3×10^-5^	0.019	0.015	1.8×10^-1^	*BCL7B*	M
rs1558902	16	0.041	0.012	6.0×10^-4^	0.012	0.010	2.3×10^-1^	*FTO*	M
rs7561317	2	0.055	0.015	1.5×10^-4^	0.013	0.012	2.9×10^-1^	*TMEM18*	M
rs6065906	20	0.026	0.014	6.6×10^-2^	0.039	0.012	1.2×10^-3^	*PLTP*	B
rs571312	18	0.038	0.013	3.5×10^-3^	0.006	0.011	6.0×10^-1^	*MC4R*	M
rs6734238	2	0.040	0.011	3.9×10^-4^	0.051	0.010	1.3×10^-7^	*IL1F10*	B

^a^ Model 1: adjusted for age

^b^ Model 2: additionally adjusted for BMI, HDL-cholesterol, LDL-cholesterol, triglycerides, total cholesterol and HbA1C

^c^ B: biological pleiotropy; M: mediated pleiotropy.

The results for the associations between the pleiotropic SNPs and the associated cardiometabolic phenotypes are presented in S4 Table. Eight SNPs were not significantly associated with the cardiometabolic phenotype in the WGHS after adjustment for multiple testing. The majority of the estimates in- or decreased slightly after adjustment for CRP. However, the estimates between *APOC1* and HbA1C, *PABPC4* and triglycerides and *BCL7B* and HDL-cholesterol decreased considerably after the adjustment for CRP.

### Pathway analysis

The results from the pathway analysis including all 13 pleiotropic genes are listed in the S5 Table. A total number of 13 canonical pathways were significantly enriched using an FDR of five percent. The top pathways included the FXR/RXR activation (p = 7.4×10^-09^), LXR/RXR activation (p = 4.6×10^-05^), Maturity Onset Diabetes of the Young (MODY) signaling (p = 7.6×10^-05^), hepatic cholestasis (p = 1.1×10^-04^) and acute phase response signaling (p = 1.3×10^-04^).

## Discussion

We observed several overlapping common genetic risk factors for cardiometabolic phenotypes and systemic inflammation. The additional analyses provided evidence for six biological pleiotropic loci with independent effects on both CRP and the cardiometabolic phenotype. These pleiotropic loci suggest a shared genetic background for CRP and cardiometabolic phenotypes. In addition, 5 pleiotropic loci appeared to have an effect on CRP mediated through the cardiometabolic phenotypes. Taken together, our results highlight the complex shared genetic architecture of cardiometabolic phenotypes and chronic inflammation.

Several of the identified biological pleiotropic loci suggest that the association between CRP and cardiometabolic phenotypes is not only reverse causation, but also shared independent genetic effects. Both the *HNF1A* and *HNF4A* loci were associated with CRP after adjustment for the cardiometabolic phenotypes. The effect directions were the same for type 2 diabetes and CRP, implying people carrying the risk allele for type 2 diabetes also have higher CRP values. We observed this also for the *PPP1R3B* locus where people carrying the risk allele for higher cholesterol also experience higher CRP levels. In both cases the effect on CRP is independent of the effect on the corresponding cardiometabolic trait.

Three of the associations that were not reported in the GWAS on CRP-level (*FTO*, *TMEM18* and *MC4R*) are associations with SNPs discovered in the GWAS on BMI by Speliotes et al. [20]. Moreover, these SNPs were also the leading findings in this large BMI GWAS meta-analysis. Our additional analyses clearly showed that after adjustment for BMI, the effects of *FTO* and *TMEM18* decreased substantially, resulting in a non-significant association, which suggests that their effect on inflammation is indeed mediated by BMI. This is in line with previous research that already provided evidence for a causal role of BMI in inflammation [14]. Conversely, none of the SNPs identified in the CRP GWAS were associated with BMI when we tested these SNPs in the BMI GWAS.

Our results also suggest a role for lipids in systemic inflammation. When we adjusted the association between *BCL7B* loci and CRP for the cardiometabolic phenotypes including lipids, the association was not present anymore. This locus appears to increase systemic inflammation through their effect on lipids. Also the association between *PABPC4* and CRP decreased after adjustment for CRP, but there was a significant residuals effect suggesting partly mediated effects through lipids. The observation that lipids may cause inflammation is in line with previous studies that have shown an important role for oxidized LDL-cholesterol molecules and free fatty acids in the development of systemic inflammation [33]. However, in addition to the mediated pleiotropic loci among lipids and CRP, we also observed loci with independent effects (biological pleiotropy) on lipids and CRP including *APOC1*, *HNF1A* and *HNF4A*, highlighting the complex interrelationship of lipids and inflammation. Moreover, the pathway analysis confirmed the role of the pleiotropic genes in both inflammation and lipid metabolism.

We observed little overlap between risk loci for CAD and CRP. Apart from the *IL6R* gene, our results suggest an association with CAD at the *HNF1A* locus. The *HNF1A* gene is an important hepatic nuclear transcription factor that has been associated in GWAS with lipids and diabetes [19,21]. This gene is known to regulate many target genes involved in lipid metabolism and transport [34]. A previous study has associated this locus with different cardiovascular phenotypes including coronary artery calcification and incident CHD [35]. Unfortunately we were not able to look-up 9 CRP SNPs in the larger CAD Metabochip GWAS because these variants were not on the Metabochip and no appropriate proxies were available. This might partly explain the little overlap between CRP and CAD genetic risk variants.

In the additional analyses we used glycated hemoglobin (HbA1C) to adjust for fasting glucose, fasting insulin, T2D and other components of the glucose homeostasis. HbA1C represents the average glucose level in the last 3 months, implying that this is only a proxy for the complex glucose homeostasis rather than a covariate that reflects its entire biological metabolism. Therefore, there may still be residual confounding from other biological pathways that have an effect on glucose and insulin levels. This could explain the observed residual effect of *GCKR* on CRP after adjustment for the cardiometabolic phenotypes.

In the evaluation of the type of pleiotropy, we adjusted the association between the pleiotropic SNP and CRP for the cardiometabolic phenotypes. For some variants we observed a convincing attenuation in the effect estimates (*BCL7B*, *FTO* and *TMEM18*). For other variants, the attenuation was less pronounced (*GCKR* and *PABPC4*). From these results we cannot conclude whether the residual effect is residual confounding or a true residual effect. Additionally, for several variants the effect estimates were the same or even increased after adjustment suggesting biological pleiotropy. The latter increase in estimate might be due to negative confounding where the SNP has an opposite direction of effect on the cross-associated phenotype compared to CRP and the effect of this phenotype on CRP is in the same direction (negative confounding). We also analyzed the association between the pleiotropic SNP and the cardiometabolic phenotypes unadjusted and adjusted for CRP. Although there is ample of evidence against a causal role for CRP in the development of cardiometabolic phenotypes, for some associations the effect estimates attenuated considerably [11–14]. This might be explained by the fact that CRP is correlated with many intermediate phenotypes that mediate the association between the SNP and the cardiometabolic phenotype.

Among some pleiotropic SNPs we observed opposite direction of effects on the phenotypes than one would expect based on their effects on the health of the possessor and the association of CRP and these cardiometabolic phenotypes in observational data. This phenomenon is known as “antagonistic pleiotropy” [36]. For instance, the SNP in the *HNF1A* locus increases serum CRP level according to the G allele and decreases LDL-cholesterol level. For biological pleiotropic loci we can substantiate this antagonistic effect. A genetic locus may have a deleterious effect on one phenotype, but an independent beneficial effect on a second phenotype. An explanation for these findings might be the fact that the effect sizes and variances explained by the genetic variants are small and therefore they only play a minor role in the phenotypical correlations. Moreover, the high frequency of seemingly detrimental alleles in human populations may partly be the effect of antagonistic pleiotropy [37]. As expected, among the loci where no independent effect was observed (mediated pleiotropy), we did not observe antagonistic pleiotropy.

Our study has certain strengths. We used the largest available GWAS data on lipids, blood pressure, BMI, CAD, glycemic traits, T2D and CRP from the GLGC, ICBP, GIANT, CARDIoGRAMplusC4D, MAGIC, DIAGRAM and CHARGE Inflammation consortia to attain as much power as possible. By including only genome-wide significant findings, we restricted the analysis to the most robust genetic associations. Moreover, we used a conservative method to correct for multiple testing, lowering the probability of false positive findings. Nonetheless, some limitations should be acknowledged. Although we used the largest available GWAS sample sizes, the identified common genetic variants for above mentioned phenotypes only explain a modest fraction of the genetic variance of these phenotypes (ranging from 5 to 12 percent). Therefore, the effects of the cardiometabolic SNPs on CRP and vice-versa may still be too small to detect cross-phenotype associations, resulting in an underestimation of the amount of genetic overlap. Moreover, we only focused on common SNPs and it might be that also rare variants underlie the shared genetic associations. The method we applied to distinguish “biological” from “mediated” pleiotropy is a classical and widely used approach in the field of epidemiology. However, we cannot completely rule out reverse causation or unknown confounders as potential drivers of the association between the genetic variant and CRP. Furthermore, we only studied GWAS including participants of European ancestry. We are aware of differences in haplotype structures between different ethnicities; however, the results are likely to be generalizable given the biological pathways.

In conclusion, we observed several genetic loci with independent effects on both CRP and one or more cardiometabolic phenotypes. These results suggest that the association between CRP and cardiometabolic phenotypes is partly explained by a shared genetic background.

## Supporting Information

S1 Table The associations of CRP SNPs with body mass index and cholesterol levels. (DOCX)

S2 Table The associations of CRP SNPs with coronary artery disease, glycaemic phenotypes and blood pressure. (DOCX)

S3 Table Pleiotropic SNPs and their association with CRP stepwise adjusted for cardiometabolic phenotypes. (DOCX)

S4 Table Pleiotropic SNPs and their association with cardiometabolic phenotype. (DOCX)

S5 Table Pathway analysis results from the 13 pleiotropic genes. (DOCX)

## References

[1] Danesh J, Wheeler JG, Hirschfield GM, Eda S, Eiriksdottir G, Rumley A, et al (2004) C-reactive protein and other circulating markers of inflammation in the prediction of coronary heart disease. N Engl J Med 350: 1387–1397. 15070788 10.1056/NEJMoa032804

[2] Sesso HD, Buring JE, Rifai N, Blake GJ, Gaziano JM, Ridker PM (2003) C-reactive protein and the risk of developing hypertension. JAMA 290: 2945–2951. 14665655 10.1001/jama.290.22.2945

[3] Dehghan A, Kardys I, de Maat MP, Uitterlinden AG, Sijbrands EJ, Bootsma AH, et al (2007) Genetic variation, C-reactive protein levels, and incidence of diabetes. Diabetes 56: 872–878. 17327459 10.2337/db06-0922

[4] Pradhan AD, Manson JE, Rifai N, Buring JE, Ridker PM (2001) C-reactive protein, interleukin 6, and risk of developing type 2 diabetes mellitus. JAMA 286: 327–334. 11466099 10.1001/jama.286.3.327

[5] Cushman M, Arnold AM, Psaty BM, Manolio TA, Kuller LH, Burke GL, et al (2005) C-reactive protein and the 10-year incidence of coronary heart disease in older men and women: the cardiovascular health study. Circulation 112: 25–31. 15983251 10.1161/CIRCULATIONAHA.104.504159

[6] Blake GJ, Rifai N, Buring JE, Ridker PM (2003) Blood pressure, C-reactive protein, and risk of future cardiovascular events. Circulation 108: 2993–2999. 14638538 10.1161/01.CIR.0000104566.10178.AF

[7] Ridker PM, Cushman M, Stampfer MJ, Tracy RP, Hennekens CH (1997) Inflammation, aspirin, and the risk of cardiovascular disease in apparently healthy men. N Engl J Med 336: 973–979. 9077376 10.1056/NEJM199704033361401

[8] Rost NS, Wolf PA, Kase CS, Kelly-Hayes M, Silbershatz H, Massaro JM, et al (2001) Plasma concentration of C-reactive protein and risk of ischemic stroke and transient ischemic attack: the Framingham study. Stroke 32: 2575–2579. 11692019 10.1161/hs1101.098151

[9] Ridker PM, Cushman M, Stampfer MJ, Tracy RP, Hennekens CH (1998) Plasma concentration of C-reactive protein and risk of developing peripheral vascular disease. Circulation 97: 425–428. 9490235 10.1161/01.cir.97.5.425

[10] Hindorff LA, Rice KM, Lange LA, Diehr P, Halder I, Walston J, et al (2008) Common variants in the CRP gene in relation to longevity and cause-specific mortality in older adults: the Cardiovascular Health Study. Atherosclerosis 197: 922–930. 17888441 10.1016/j.atherosclerosis.2007.08.012 PMC2362133

[11] Elliott P, Chambers JC, Zhang W, Clarke R, Hopewell JC, Peden JF, et al (2009) Genetic Loci associated with C-reactive protein levels and risk of coronary heart disease. JAMA 302: 37–48. 10.1001/jama.2009.954 19567438 PMC2803020

[12] Collaboration CRPCHDG (2011) Association between C reactive protein and coronary heart disease: mendelian randomisation analysis based on individual participant data. BMJ: British Medical Journal 342. 10.1136/bmj.d548 PMC3039696 21325005

[13] Smith GD, Lawlor DA, Harbord R, Timpson N, Rumley A, Lowe GDO, et al (2005) Association of C-reactive protein with blood pressure and hypertension life course confounding and Mendelian randomization tests of causality. Arteriosclerosis, thrombosis, and vascular biology 25: 1051–1056. 15731495 10.1161/01.ATV.0000160351.95181.d0

[14] Timpson NJ, Nordestgaard BG, Harbord RM, Zacho J, Frayling TM, Tybjærg-Hansen A, et al (2011) C-reactive protein levels and body mass index: elucidating direction of causation through reciprocal Mendelian randomization. International Journal of Obesity 35: 300–308. 10.1038/ijo.2010.137 20714329 PMC4783860

[15] The CDC, Deloukas P, Kanoni S, Willenborg C, Farrall M, Assimes TL, et al (2012) Large-scale association analysis identifies new risk loci for coronary artery disease. Nat Genet 45: 25–33. 10.1038/ng.2480 23202125 PMC3679547

[16] International Consortium for Blood Pressure Genome-Wide Association S, Ehret GB, Munroe PB, Rice KM, Bochud M, Johnson AD, et al (2011) Genetic variants in novel pathways influence blood pressure and cardiovascular disease risk. Nature 478: 103–109. 10.1038/nature10405 21909115 PMC3340926

[17] Dupuis J, Langenberg C, Prokopenko I, Saxena R, Soranzo N, Jackson AU, et al (2010) New genetic loci implicated in fasting glucose homeostasis and their impact on type 2 diabetes risk. Nat Genet 42: 105–116. 10.1038/ng.520 20081858 PMC3018764

[18] Scott RA, Lagou V, Welch RP, Wheeler E, Montasser ME, Luan J, et al (2012) Large-scale association analyses identify new loci influencing glycemic traits and provide insight into the underlying biological pathways. Nat Genet 44: 991–1005. 10.1038/ng.2385 22885924 PMC3433394

[19] Morris AP, Voight BF, Teslovich TM, Ferreira T, Segre AV, Steinthorsdottir V, et al (2012) Large-scale association analysis provides insights into the genetic architecture and pathophysiology of type 2 diabetes. Nat Genet 44: 981–990. 10.1038/ng.2383 22885922 PMC3442244

[20] Speliotes EK, Willer CJ, Berndt SI, Monda KL, Thorleifsson G, Jackson AU, et al (2010) Association analyses of 249,796 individuals reveal 18 new loci associated with body mass index. Nat Genet 42: 937–948. 10.1038/ng.686 20935630 PMC3014648

[21] Teslovich TM, Musunuru K, Smith AV, Edmondson AC, Stylianou IM, Koseki M, et al (2010) Biological, clinical and population relevance of 95 loci for blood lipids. Nature 466: 707–713. 10.1038/nature09270 20686565 PMC3039276

[22] Dehghan A, Dupuis J, Barbalic M, Bis JC, Eiriksdottir G, Lu C, et al (2011) Meta-analysis of genome-wide association studies in >80 000 subjects identifies multiple loci for C-reactive protein levels. Circulation 123: 731–738. 10.1161/CIRCULATIONAHA.110.948570 21300955 PMC3147232

[23] Solovieff N, Cotsapas C, Lee PH, Purcell SM, Smoller JW (2013) Pleiotropy in complex traits: challenges and strategies. Nat Rev Genet 14: 483–495. 10.1038/nrg3461 23752797 PMC4104202

[24] Olden M, Teumer A, Bochud M, Pattaro C, Köttgen A, Turner ST, et al (2013) Overlap between common genetic polymorphisms underpinning kidney traits and cardiovascular disease phenotypes: the CKDGen Consortium. American Journal of Kidney Diseases 61: 889–898. 10.1053/j.ajkd.2012.12.024 23474010 PMC3660426

[25] Johnson AD, Handsaker RE, Pulit SL, Nizzari MM, O’Donnell CJ, de Bakker PIW (2008) SNAP: a web-based tool for identification and annotation of proxy SNPs using HapMap. Bioinformatics 24: 2938–2939. 10.1093/bioinformatics/btn564 18974171 PMC2720775

[26] Voight BF, Kang HM, Ding J, Palmer CD, Sidore C, Chines PS, et al (2012) The metabochip, a custom genotyping array for genetic studies of metabolic, cardiovascular, and anthropometric traits. PLoS Genet 8: e1002793 10.1371/journal.pgen.1002793 22876189 PMC3410907

[27] Schunkert H, Konig IR, Kathiresan S, Reilly MP, Assimes TL, Holm H, et al (2011) Large-scale association analysis identifies 13 new susceptibility loci for coronary artery disease. Nat Genet 43: 333–338. 10.1038/ng.784 21378990 PMC3119261

[28] McIntyre LM, Martin ER, Simonsen KL, Kaplan NL (2000) Circumventing multiple testing: a multilocus Monte Carlo approach to testing for association. Genet Epidemiol 19: 18–29. 10861894 10.1002/1098-2272(200007)19:1<18::AID-GEPI2>3.0.CO;2-Y

[29] Peng G, Luo L, Siu H, Zhu Y, Hu P, Hong S, et al (2010) Gene and pathway-based second-wave analysis of genome-wide association studies. European Journal of Human Genetics 18: 111–117. 10.1038/ejhg.2009.115 19584899 PMC2987176

[30] Ridker PM, Chasman DI, Zee RY, Parker A, Rose L, Cook NR, et al (2008) Rationale, design, and methodology of the Women’s Genome Health Study: a genome-wide association study of more than 25,000 initially healthy american women. Clin Chem 54: 249–255. 18070814 10.1373/clinchem.2007.099366

[31] Nathan DM, Singer DE, Hurxthal K, Goodson JD (1984) The clinical information value of the glycosylated hemoglobin assay. N Engl J Med 310: 341–346. 6690962 10.1056/NEJM198402093100602

[32] Team RC (2012) R: A language and environment for statistical computing R Foundation for Statistical Computing, Vienna, Austria Available from: http://wwwR-projectorg/.

[33] Rocha VZ, Libby P (2009) Obesity, inflammation, and atherosclerosis. Nat Rev Cardiol 6: 399–409. 10.1038/nrcardio.2009.55 19399028

[34] Odom DT, Zizlsperger N, Gordon DB, Bell GW, Rinaldi NJ, Murray HL, et al (2004) Control of pancreas and liver gene expression by HNF transcription factors. Science 303: 1378–1381. 14988562 10.1126/science.1089769 PMC3012624

[35] Reiner AP, Gross MD, Carlson CS, Bielinski SJ, Lange LA, Fornage M, et al (2009) Common Coding Variants of the HNF1A Gene Are Associated With Multiple Cardiovascular Risk Phenotypes in Community-Based Samples of Younger and Older European-American Adults The Coronary Artery Risk Development in Young Adults Study and The Cardiovascular Health Study. Circulation: Cardiovascular Genetics 2: 244–254. 10.1161/CIRCGENETICS.108.839506 20031592 PMC2841292

[36] Williams GC (2001) Pleiotropy, natural selection, and the evolution of senescence. Science’s SAGE KE 2001: 13.

[37] Carter AJ, Nguyen AQ (2011) Antagonistic pleiotropy as a widespread mechanism for the maintenance of polymorphic disease alleles. BMC Med Genet 12: 160 10.1186/1471-2350-12-160 22151998 PMC3254080

