# Supplementary material for: Pleiotropy among Common Genetic Loci Identified for Cardiometabolic Disorders and C-Reactive Protein
Source: PLoS One. 2015 Mar 13;10(3):e0118859. doi: 10.1371/journal.pone.0118859 (PMC4358943; doi:10.1371/journal.pone.0118859)
Supplement: S1 Table — (DOCX) [file pone.0118859.s001.docx]

**S1a Table. The associations of CRP SNPs with body mass index and cholesterol levels.**

| **SNP** | **Gene** | **A1^*^** | **CRP** | **BMI** | **TC** | **HDLC** | **LDLC** | **TG** |
| --- | --- | --- | --- | --- | --- | --- | --- | --- |
| rs2794520 | *CRP* | C | 9.5×10^-189^(+) | 0.53(-) | 0.59(+) | 0.77(-) | 0.35(+) | 0.66(-) |
| rs4420638 | *APOC1* | A | 2.1×10^-129^(+) | 0.25(+) | **5.2×10^-111^**(-) | **4.4×10^-21^**(+) | **8.7×10^-147^**(-) | **5.4×10^-22^**(-) |
| rs1183910 | *HNF1A* | G | 3.3×10^-113^(+) | 0.86(+) | **5.2×10^-14^**(-) | 4.0×10^-3^(-) | **5.8×10^-15^**(-) | 0.58(-) |
| rs4420065 | *LEPR* | C | 3.2×10^-64^(+) | 0.36(-) | 0.08(-) | 0.09(-) | 0.38(-) | 0.18(-) |
| rs4129267 | *IL6R* | C | 1.1×10^-47^(+) | 0.62(-) | 0.68(-) | 0.90(-) | 0.80(-) | 0.84(-) |
| rs1260326 | *GCKR* | T | 5.4×10^-43^(+) | 0.13(-) | **7.3×10^-27^**(+) | 0.08(-) | 2.2×10^-4^(+) | **5.7×10^-133^**(+) |
| rs12239046 | *NLRP3* | C | 1.6×10^-13^(+) | 0.92(-) | 0.37(-) | 0.04(-) | 0.95(+) | 0.30(-) |
| rs6734238 | *IL1F10* | G | 3.4×10^-13^(+) | 0.74(-) | **1.2×10^-5^**(-) | 0.18(-) | 6.5×10^-3^(-) | 0.03(-) |
| rs9987289 | *PPP1R3B* | G | 2.3×10^-12^(+) | 0.39(-) | **7.1×10^-23^**(+) | **6.4×10^-25^**(+) | **2.0×10^-14^**(+) | 0.02(-) |
| rs10745954 | *ASCL1* | A | 1.6×10^-11^(+) | 0.48(-) | 0.66(-) | 0.10(+) | 0.03(-) | 1.6×10^-3^(+) |
| rs1800961 | *HNF4A* | C | 2.3×10^-11^(+) | 0.91(-) | **5.7×10^-13^**(+) | **1.1×10^-15^**(+) | **2.4×10^-5^**(+) | 0.59(+) |
| rs340029 | *RORA* | T | 2.6×10^-11^(+) | 0.01(+) | 0.01(-) | 0.67(+) | 3.8×10^-3^(-) | 0.74(+) |
| rs10521222 | *SALL1* | C | 1.3×10^-10^(+) | 0.72(+) | 0.57(-) | 0.65(+) | 0.58(-) | 0.20(-) |
| rs12037222 | *PABPC4* | A | 4.5×10^-10^(+) | 0.22(+) | 0.07(+) | **1.6×10^-9^**(-) | 8.7×10^-3^(+) | **7.0×10^-7^**(+) |
| rs4705952 | *IRF1* | G | 1.3×10^-8^(+) | 0.37(+) | 4.1x10^-3^(-) | 0.56(-) | 3.2x10^-3^(-) | 0.52(+) |
| rs2847281 | *PTPN2* | A | 2.2×10^-8^(+) | 2.73x10^-3^(-) | 0.77(+) | 0.15(+) | 0.64(-) | 0.13(-) |
| rs13233571 | *BCL7B* | C | 2.8×10^-8^(+) | 0.66(+) | 0.12(+) | **2.9×10^-9^**(-) | 0.14(-) | **9.3×10^-58^**(+) |
| rs6901250 | *GPRC6A* | A | 4.8×10^-8^(+) | 0.66(+) | 0.08(+) | 0.02(+) | 0.13(+) | 0.25(-) |

^*^ A1 represents the risk allele according to the CRP GWAS.

*Note*: p-value ≤ 1.1×10^-4^ is considered as study-wide significant (0.05/463).

Abbreviations: BMI, body mass index; CRP, c-reactive protein; HDLC, HDL-cholesterol; LDLC, LDL-cholesterol; SNP, single-nucleotide polymorphism; TC, total cholesterol; TG, triglycerides.
