# Supplementary material for: Pleiotropy among Common Genetic Loci Identified for Cardiometabolic Disorders and C-Reactive Protein
Source: PLoS One. 2015 Mar 13;10(3):e0118859. doi: 10.1371/journal.pone.0118859 (PMC4358943; doi:10.1371/journal.pone.0118859)
Supplement: S2 Table — (DOCX) [file pone.0118859.s002.docx]

**S1b Table. The associations of CRP SNPs with coronary artery disease, glycaemic phenotypes and blood pressure.**

| **SNP** | **Gene** | **A1^*^** | **CRP** | **CAD** | **FG** | **FI** | **T2D** | **SBP** |
| --- | --- | --- | --- | --- | --- | --- | --- | --- |
| rs2794520 | *CRP* | C | 9.5×10^-189^(+) | 0.66(-) | 0.39(-) | 0.32(-) | 0.29 (-) | 4.0×10^-3^(+) |
| rs4420638 | *APOC1* | A | 2.1×10^-129^(+) | 2.1×10^-4^(-) | 3.1×10^-4^ (+) | 0.03(+) | **4.0×10^-6^** (-) | 0.30(+) |
| rs1183910 | *HNF1A* | G | 3.3×10^-113^(+) | **5.6×10^-6^**(-) | 0.29(-) | 0.02(+) | 2.0×10^-4^ (+) | 0.46(-) |
| rs4420065 | *LEPR* | C | 3.2×10^-64^(+) | 0.04(-) | 0.57(+) | 0.77(-) | 0.94(+) | 0.39(+) |
| rs4129267 | *IL6R* | C | 1.1×10^-47^(+) | **1.7×10^-8^**(+) | 0.70(-) | 0.88(-) | 0.26 (-) | 0.50(+) |
| rs1260326 | *GCKR* | T | 5.4×10^-43^(+) | 0.84(-) | **2.17×10^-41^**(-) | **3.8×10^-14^(-)** | **1.6×10^-6^** (-) | 0.30(+) |
| rs12239046 | *NLRP3* | C | 1.6×10^-13^(+) | 0.73(+) | 0.15(+) | 0.97(-) | 0.47(-) | 0.54(-) |
| rs6734238 | *IL1F10* | G | 3.4×10^-13^(+) | 0.02(-) | 5.8×10^-3^(-) | 0.24(+) | 0.62(+) | 0.77(-) |
| rs9987289 | *PPP1R3B* | G | 2.3×10^-12^(+) | 0.81(-) | **6.1×10^-13^**(-) | **1.1×10^-11^**(-) | 3.7×10^-3^ (+) | 0.58(-) |
| rs10745954 | *ASCL1* | A | 1.6×10^-11^(+) | 0.08(-) | 0.71(+) | 0.62(-) | 0.79 (+) | 0.81(+) |
| rs1800961 | *HNF4A* | C | 2.3×10^-11^(+) | 0.27(-) | 0.15(+) | 0.25(-) | 2.7×10^-4^ (+) | 0.54(+) |
| rs340029 | *RORA* | T | 2.6×10^-11^(+) | 0.61(-) | 0.67(-) | 0.59(+) | 0.10(+) | 0.85(+) |
| rs10521222 | *SALL1* | C | 1.3×10^-10^(+) | 0.58(+) | 0.83(+) | 0.40(+) | 0.68(-) | 0.36(-) |
| rs12037222 | *PABPC4* | A | 4.5×10^-10^(+) | 0.86(-) | 0.03(+) | 0.10(+) | 4.9×10^-3^ (+) | 3.7×10^-3^(+) |
| rs4705952 | *IRF1* | G | 1.3×10^-8^(+) | 0.26(-) | 1.7×10^-4^(-) | 0.14(-) | 0.68(+) | 0.08(-) |
| rs2847281 | *PTPN2* | A | 2.2×10^-8^(+) | 0.43(-) | 0.02(-) | 0.90(-) | 0.29(+) | 0.16(+) |
| rs13233571 | *BCL7B* | C | 2.8×10^-8^(+) | 0.08(-) | 0.09(-) | 0.02(-) | 8.3×10^-4^ (+) | 0.49(+) |
| rs6901250 | *GPRC6A* | A | 4.8×10^-8^(+) | 0.85(+) | 0.30(+) | 0.06(-) | 0.15(-) | 0.47(-) |

^*^ A1 represents the risk allele according to the CRP GWAS.

*Note*: p-value ≤ 1.1×10^-4^ is considered as study-wide significant (0.05/463).

Abbreviations: CRP, C-reactive protein; CAD, coronary artery disease; FG, fasting glucose; FI, fasting insulin; SBP, systolic blood pressure; SNP, single-nucleotide polymorphism; T2D, type 2 diabetes.
