# Supplementary material for: Pleiotropy among Common Genetic Loci Identified for Cardiometabolic Disorders and C-Reactive Protein
Source: PLoS One. 2015 Mar 13;10(3):e0118859. doi: 10.1371/journal.pone.0118859 (PMC4358943; doi:10.1371/journal.pone.0118859)
Supplement: S3 Table — (DOCX) [file pone.0118859.s003.docx]

**S3 Table. Pleiotropic SNPs and their association with CRP stepwise adjusted for cardiometabolic phenotypes.**

|  |  | **Model 1^a^** | | | **Model 2^b^** | | | **Model 3^c^** | | | **Model 4**^d^ | | |
| --- | --- | --- | --- | --- | --- | --- | --- | --- | --- | --- | --- | --- | --- |
| **SNP** | **gene** | **beta** | **se** | **pval** | **beta** | **se** | **pval** | **beta** | **se** | **pval** | **beta** | **se** | **pval** |
| rs4420638 | *APOC1* | 0.269 | 0.019 | 4.4×10^-47^ | 0.310 | 0.017 | 1.3×10^-72^ | 0.276 | 0.016 | 8.2×10^-68^ | 0.272 | 0.016 | 1.7×10^-65^ |
| rs1169288 | *HNF1A* | 0.165 | 0.012 | 2.3×10^-43^ | 0.164 | 0.011 | 1.0×10^-50^ | 0.161 | 0.010 | 5.9×10^-57^ | 0.160 | 0.010 | 4.0×10^-56^ |
| rs1260326 | *GCKR* | 0.110 | 0.011 | 1.6×10^-22^ | 0.052 | 0.010 | 7.2×10^-07^ | 0.073 | 0.010 | 3.5×10^-14^ | 0.073 | 0.010 | 3.4×10^-14^ |
| rs4845625 | *IL6R* | 0.067 | 0.011 | 2.0×10^-9^ | 0.065 | 0.010 | 1.9×10^-10^ | 0.064 | 0.009 | 1.7×10^-11^ | 0.065 | 0.009 | 8.8×10^-12^ |
| rs9987289 | *PPP1R3B* | 0.076 | 0.019 | 4.5×10^-5^ | 0.099 | 0.018 | 2.0×10^-08^ | 0.085 | 0.016 | 2.3×10^-07^ | 0.086 | 0.016 | 1.5×10^-7^ |
| rs1800961 | *HNF4A* | 0.146 | 0.033 | 8.4×10^-6^ | 0.154 | 0.030 | 3.1×10^-07^ | 0.143 | 0.028 | 3.1×10^-07^ | 0.141 | 0.028 | 4.7×10^-7^ |
| rs4660293 | *PABPC4* | 0.048 | 0.013 | 1.9×10^-4^ | 0.031 | 0.012 | 1.0×10^-02^ | 0.037 | 0.011 | 8.2×10^-04^ | 0.036 | 0.011 | 1.2×10^-3^ |
| rs17145738 | *BCL7B* | 0.075 | 0.017 | 1.3×10^-5^ | 0.012 | 0.016 | 4.3×10^-01^ | 0.020 | 0.015 | 1.7×10^-01^ | 0.019 | 0.015 | 1.8×10^-1^ |
| rs1558902 | *FTO* | 0.041 | 0.012 | 6.0×10^-4^ | 0.034 | 0.011 | 1.5×10^-03^ | 0.011 | 0.010 | 2.7×10^-01^ | 0.012 | 0.010 | 2.3×10^-1^ |
| rs7561317 | *TMEM18* | 0.055 | 0.015 | 1.5×10^-4^ | 0.044 | 0.013 | 9.4×10^-04^ | 0.014 | 0.012 | 2.7×10^-01^ | 0.013 | 0.012 | 2.9×10^-1^ |
| rs6065906 | *PLTP* | 0.026 | 0.014 | 6.6×10^-2^ | 0.061 | 0.013 | 2.0×10^-06^ | 0.038 | 0.012 | 1.7×10^-03^ | 0.039 | 0.012 | 1.2×10^-3^ |
| rs571312 | *MC4R* | 0.038 | 0.013 | 3.5×10^-3^ | 0.024 | 0.012 | 4.6×10^-02^ | 0.004 | 0.011 | 6.9×10^-01^ | 0.006 | 0.011 | 6.0×10^-1^ |
| rs6734238 | *IL1F10* | 0.040 | 0.011 | 3.9×10^-4^ | 0.047 | 0.010 | 5.0×10^-06^ | 0.051 | 0.010 | 1.0×10^-07^ | 0.051 | 0.010 | 1.3×10^-7^ |

^a^ Model 1: adjusted for age

^b^ Model 2: adjusted for age and lipids (HDL-cholesterol, LDL-cholesterol, triglycerides and total cholesterol)

^c^ Model 3: adjusted for age, lipids and BMI

^d^ Model 4: adjusted for age, lipids, BMI and HbA1C
