# Supplementary material for: Pleiotropy among Common Genetic Loci Identified for Cardiometabolic Disorders and C-Reactive Protein
Source: PLoS One. 2015 Mar 13;10(3):e0118859. doi: 10.1371/journal.pone.0118859 (PMC4358943; doi:10.1371/journal.pone.0118859)
Supplement: S4 Table — (DOCX) [file pone.0118859.s004.docx]

**S4 Table. Pleiotropic SNPs and their association with cardiometabolic phenotype.**

|  | | *Age adjusted* | | | *Age + CRP adjusted* | | |  |  |
| --- | --- | --- | --- | --- | --- | --- | --- | --- | --- |
| **SNP** | **chr** | **beta** | **se** | **pval** | **beta** | **se** | **pval** | **gene** | **Phenotype** |
| rs4420638 | 19 | 7.657 | 0.642 | 9.5×10^-33^ | 9.047 | 0.637 | 1.4×10^-45^ | *APOC1* | TC |
| rs4420638 | 19 | 0.053 | 0.008 | 2.5×10^-10^ | 0.103 | 0.008 | 1.4×10^-39^ | *APOC1* | TG |
| rs4420638 | 19 | -1.545 | 0.236 | 6.0×10^-11^ | -2.131 | 0.233 | 7.6×10^-20^ | *APOC1* | HDLC |
| rs4420638 | 19 | 8.287 | 0.526 | 1.6×10^-55^ | 8.875 | 0.527 | 3.4×10^-63^ | *APOC1* | LDLC |
| rs4420638 | 19 | -0.026 | 0.009 | 4.2×10^-03^ | -0.006 | 0.009 | 5.0×10^-01^ | *APOC1* | HbA1C |
| rs1169288 | 12 | 2.471 | 0.411 | 1.9×10^-09^ | 3.299 | 0.409 | 7.2×10^-16^ | *HNF1A* | TC |
| rs1169288 | 12 | 2.261 | 0.338 | 2.4×10^-11^ | 2.594 | 0.339 | 2.0×10^-14^ | *HNF1A* | LDLC |
| rs1169288 | 12 | 0.003 | 0.006 | 6.4×10^-01^ | 0.017 | 0.006 | 3.5×10^-03^ | *HNF1A* | HbA1C |
| rs1260326 | 2 | 3.595 | 0.388 | 2.2×10^-20^ | 3.070 | 0.385 | 1.7×10^-15^ | *GCKR* | TC |
| rs1260326 | 2 | 0.070 | 0.005 | 8.5×10^-43^ | 0.050 | 0.005 | 7.5×10^-27^ | *GCKR* | TG |
| rs1260326 | 2 | -0.013 | 0.006 | 1.7×10^-02^ | -0.023 | 0.005 | 2.5×10^-05^ | *GCKR* | HbA1C |
| rs9987289 | 8 | -2.773 | 0.664 | 3.0×10^-05^ | -2.405 | 0.658 | 2.6×10^-04^ | *PPP1R3B* | TC |
| rs9987289 | 8 | -1.085 | 0.244 | 8.5×10^-06^ | -1.246 | 0.240 | 2.2×10^-07^ | *PPP1R3B* | HDLC |
| rs9987289 | 8 | -2.210 | 0.546 | 5.2×10^-05^ | -2.068 | 0.545 | 1.5×10^-04^ | *PPP1R3B* | LDLC |
| rs9987289 | 8 | 0.015 | 0.010 | 1.1×10^-01^ | 0.021 | 0.009 | 2.3×10^-02^ | *PPP1R3B* | HbA1C |
| rs1800961 | 20 | -4.927 | 1.130 | 1.3×10^-05^ | -4.217 | 1.119 | 1.6×10^-04^ | *HNF4A* | TC |
| rs1800961 | 20 | -2.163 | 0.415 | 1.8×10^-07^ | -2.474 | 0.409 | 1.5×10^-09^ | *HNF4A* | HDLC |
| rs1800961 | 20 | -2.960 | 0.929 | 1.4×10^-03^ | -2.685 | 0.928 | 3.8×10^-03^ | *HNF4A* | LDLC |
| rs1800961 | 20 | 0.022 | 0.016 | 1.7×10^-01^ | 0.030 | 0.016 | 6.3×10^-02^ | *HNF4A* | HbA1C |
| rs4660293 | 1 | -0.575 | 0.164 | 4.6×10^-04^ | -0.473 | 0.162 | 3.5×10^-03^ | *PABPC4* | HDLC |
| rs4660293 | 1 | 0.020 | 0.006 | 7.5×10^-04^ | 0.011 | 0.005 | 3.9×10^-02^ | *PABPC4* | TG |
| rs17145738 | 7 | 0.458 | 0.218 | 3.5×10^-02^ | 0.299 | 0.215 | 1.6×10^-01^ | *BCL7B* | HDLC |
| rs17145738 | 7 | -0.074 | 0.008 | 3.7×10^-21^ | -0.060 | 0.007 | 4.6×10^-17^ | *BCL7B* | TG |
| rs1558902 | 16 | 0.541 | 0.049 | 5.0×10^-28^ | 0.470 | 0.044 | 9.7×10^-27^ | *FTO* | BMI |
| rs1558902 | 16 | 0.028 | 0.006 | 2.2×10^-06^ | 0.025 | 0.006 | 1.9×10^-05^ | *FTO* | HbA1C |
| rs7561317 | 2 | -0.344 | 0.060 | 1.3×10^-08^ | -0.243 | 0.054 | 6.6×10^-06^ | *TMEM18* | BMI |
| rs6065906 | 20 | -1.154 | 0.178 | 9.0×10^-11^ | -1.209 | 0.175 | 5.7×10^-12^ | *PLTP* | HDLC |
| rs6065906 | 20 | 0.039 | 0.006 | 1.4×10^-09^ | 0.043 | 0.006 | 1.5×10^-13^ | *PLTP* | TG |
| rs571312 | 18 | 0.281 | 0.055 | 2.8×10^-07^ | 0.209 | 0.049 | 1.7×10^-05^ | *MC4R* | BMI |
| rs6734238 | 2 | -1.164 | 0.388 | 2.7×10^-03^ | -1.358 | 0.385 | 4.1×10^-04^ | *IL1F10* | TC |

Abbreviations: BMI, body mass index; chr, chromosome; CRP, C-reactive protein; HbA1C, haemoglobin A1C; HDLC,

HDL-cholesterol; LDLC, LDL-cholesterol; se, standard error; SNP, single-nucleotide polymorphism; TC, total cholesterol; TG, triglycerides
