# Supplementary material for: Pleiotropy among Common Genetic Loci Identified for Cardiometabolic Disorders and C-Reactive Protein
Source: PLoS One. 2015 Mar 13;10(3):e0118859. doi: 10.1371/journal.pone.0118859 (PMC4358943; doi:10.1371/journal.pone.0118859)
Supplement: S5 Table — (DOCX) [file pone.0118859.s005.docx]

**S5 Table. Pathway analysis results from the 13 pleiotropic genes.**

| **Caninocal Pathway** | **p-value^a^** |
| --- | --- |
| FXR/RXR Activation | 7.4×10^-09^ |
| LXR/RXR Activation | 4.6×10^-05^ |
| Maturity Onset Diabetes of the Young (MODY) signaling | 7.6×10^-05^ |
| Hepatic Cholestasis | 1.1×10^-04^ |
| Acute Phase Response signaling | 1.3×10^-04^ |
| LPS/IL-1 Mediated Inhibition of RXR function | 2.6×10^-04^ |
| Role of Macrophages, Ficorblasts and Endothelial Cells in Rheumatoid Arthritis | 6.6×10^-04^ |
| IL-6 signaling | 2.1×10^-03^ |
| Atherosclerosis Signaling | 2.4×10^-03^ |
| Acyl-CoA Hydrolysis | 7.2×10^-03^ |
| Role of Osetoblasts, Osteoclasts and Chondrocytes in Rheumatoid Arthritis | 7.4×10^-03^ |
| Systemic Lupus Erythematosus Signaling | 7.5×10^-03^ |
| Colorectal Cancer Metastasis Signaling | 8.5×10^-03^ |

^a^Significant at False Discovery Rate of 5 percent.
